# New Data on the Distribution and Systematic Position of *Marsupella minutissima*, a Poorly Known Species from Gymnomitriaceae (Marchantiophyta)

**DOI:** 10.3390/plants13213037

**Published:** 2024-10-30

**Authors:** Vadim A. Bakalin, Seung Se Choi, Vladimir E. Fedosov, Myung-Ok Moon, Alina V. Fedorova

**Affiliations:** 1 Laboratory of Cryptogamic Biota, Botanical Garden-Institute FEB RAS, Makovskogo Street 142, Vladivostok 690024, Russia; fedosov_v@mail.ru; 2 Team of National Ecosystem Survey, National Institute of Ecology, Seocheon 33657, Republic of Korea; 3 Faculty of Biology, Lomonosov Moscow State University, Leninskie Gory Str. 1–12, Moscow 119234, Russia; 4 Institute of Forestry, Jeju 63133, Republic of Korea; egosari@naver.com; 5 Laboratory of Molecular Systematics of Plants, Tsitsin Main Botanical Garden, Russian Academy of Sciences, Botanicheskaya Str., 4, Moscow 127276, Russia; alina_77777@mail.ru

**Keywords:** East Asia, Korea, ITS, trnL-F, Jeju-do, liverworts

## Abstract

*Marsupella minutissima* was recorded for the first time outside of Japan in Jeju-do in the Republic of Korea. The new data significantly improve the knowledge of the species distribution. An analysis of the phylogenetic affinities of the species based on nuclear ITS and plastid *trn*L-*trn*F regions showed that it is resolved within the clade corresponding to *Marsupella* sect. *Ustulatae*, which is where the most morphologically similar *M. funckii* and *M. sprucei* are placed. These tree taxa are well delimited geographically. When found within the same region, such as a mountain chain, they occupy different altitudinal belts. Our results showed clear molecular delimitation; however, we failed to estimate the affinities of *M. minutissima* within the section based on the available molecular data. A description, line-art illustrations and photographs of the collected materials are provided; data on the oil bodies of the species were obtained for the first time.

## 1. Introduction

Although *Marsupella* has received much attention in the last decade, not all species of the genus are well studied, thus new information on distribution and species diversity is published regularly [1,2,3,4,5]. Nevertheless, *M. alata* S.Hatt & N.Kitag., *M. apertifolia* Steph., *M. bolanderi* (Austin) Underw., *M. patens* (N.Kitag.) Bakalin & Fedosov, *M. microphylla* R.M.Schust. and many other species remain understudied. At least 50% of the species in the genus are insufficiently understood. One such poorly known taxon is *M. minutissima*, which, to date, has been found only in Japan. The species was described by Kitagawa [6] from Mt. Nishiakaishi in the Ehime Prefecture and was later found in the Tottori Prefecture on Honshu [7], both of which are located in the middle of western Japan. When small *Marsupella* plants were discovered on Hallasan Mountain on Jeju Island in the Republic of Korea, this particular taxon was suspected based on the identification key placed in Kitagawa [7]. Further microscopic investigation and a review of the type of species in KYO confirmed the identity, as described below.

The position of *Marsupella minutissima* in the *Marsupella* phylogeny remains unclear, as it has never been included in phylogenetic analyses. Kitagawa [7] classified this species within the sect. *Marsupella*, although with doubt (the position in his list is marked with a question mark). This classification is somewhat unexpected, since Kitagawa [6] showed its morphological similarity with *Marsupella sprucei*, which he attributed to the sect. *Funkiae* Müll.Frib. (=sect. *Ustulatae* R.M. Schust.). Notably, Kitagawa placed within the latter section also *M. commutata* (Limpr.) Bernet (=*Gymnomitrion commutatum* (Limpr.) Schiffn.) and *Marsupella alpina* (Gottsche ex Husn.) Bernet (=*Gymnomitrion alpinum* (Gottsche ex Husn.) Schiffn.). However, both the latter were classified to another genus (*Gymnomitrion*), supported by the molecular phylogenetic data [8,9]. The same rather controversial point of view on the position of *M. minutissima* within the sect. *Marsupella* was adopted by Bakalin et al. [2], with similar doubts regarding the uncertainty of the species’ position. Dramatic changes in the understanding of two morphologically overlapping genera, *Gymnomitrion* and *Marsupella,* have occurred since the beginning of molecular phylogenetic studies of this group [8,9]. The morphology of *Marsupella* was found malleable even within the same section; for example, some species, *e.g*. in *Marsupella* sect. *Stolonicaulon* (N.Kitag.) Váňa, show superficial morphological similarities with representatives of various families, cf. Bakalin et al., [3]. It is expected that the recently collected material may provide new understanding of the *Marsupella* phylogeny.

Considering the state of knowledge on the *Marsupella* system as well as the availability of material, the main goals of this work were to provide a comprehensive description of the collected plants and determine their position in the phylogenetic system of the genus.

## 2. Results

Based on a morphology comparison of collected specimens with available type material and descriptions [6,7], *Marsupella minutissima* is revealed for the first time in the Republic of Korea on Jeju-do. The present report is the first confirmed case outside of Japan. In the Catalogue of Life: 2019 Annual Checklist (https://www.catalogueoflife.org/annual-checklist/2019/details/species/id/6aab3fc0ed17c1262396199e610e38a8 accessed on 15 March 2024), the species was reported in the Fujian Province of China, but the original source of this report could not be located. The species is also lacking in the list of liverworts of this province [10].

**Figure 1:**
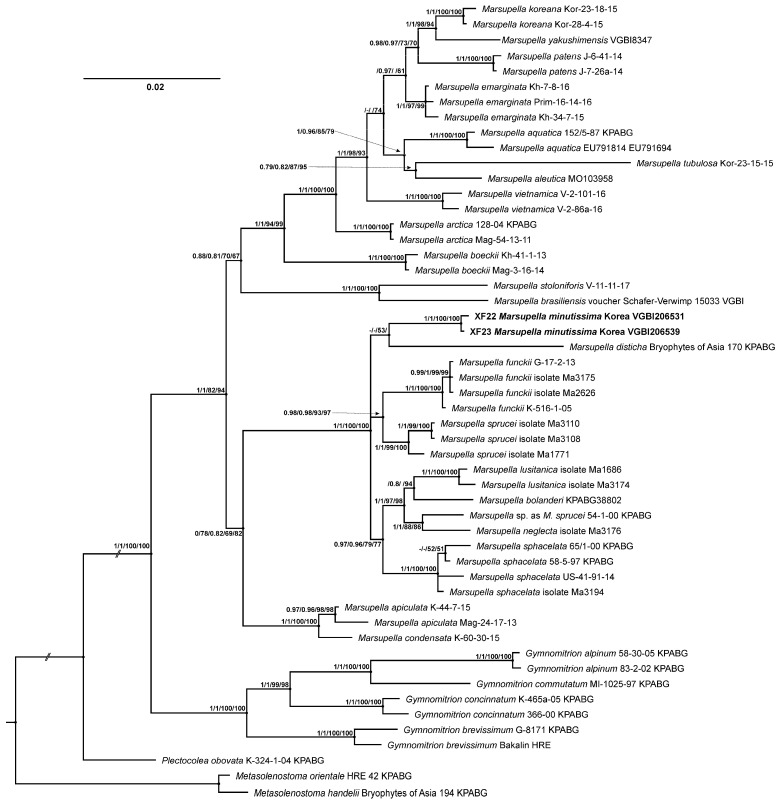
Bayesian tree of a selection of *Marsupella* specimens, with particular focus on the sect. *Ustulatae*, inferred from combined sequences of cp trnL-trnF region and nuclear ITS. Posterior probabilities of ≥0.7 obtained from the analyses without and with indel data and bootstrap values inferred from ML values of ≥50 (without and with indel data) are shown above the branches. Dashes indicate lower support, while spaces indicate lack of nodes in topology, as inferred from a particular analysis. The newly obtained sequences are in bold.

In the trees obtained from the combined nr ITS and plastid *trn*L-F dataset, the clades corresponding to species level typically have high to maximal statistical support, whereas the resolution and support of the deeper nodes remains rather weak. The data from molecular phylogenetic analyses revealed that *M. minutissima* is resolved as a member of the sect. *Marsupella* sect. *Ustulatae*, which is far from the section where it was previously placed (Figure 1). This newly revealed clade does not support grouping with any of the previously studied species, and is neither morphologically nor phytogeographically close.

Since our study did not conduct a molecular analysis of Japanese materials, it is unknown if the Japanese populations differ from the Korean populations genetically. Minor morphological differences may be observed, as discussed below. To prevent compiling the mixture of morphological features below, a morphological description of the species based entirely on the Korean materials is provided.

*Marsupella minutissima* N.Kitag., Mem. Coll. Sci. Kyoto Imp. Univ., Ser. B, Biol. 27: 81, 1960, Mt. Nishiakaishi, Ehime Prefecture, Shikoku, Japan, leg K. Oti-1927 1 June 1947 (Holotype—NICH, not seen, isotype KYO, s.n.!).

**Figure 2:**
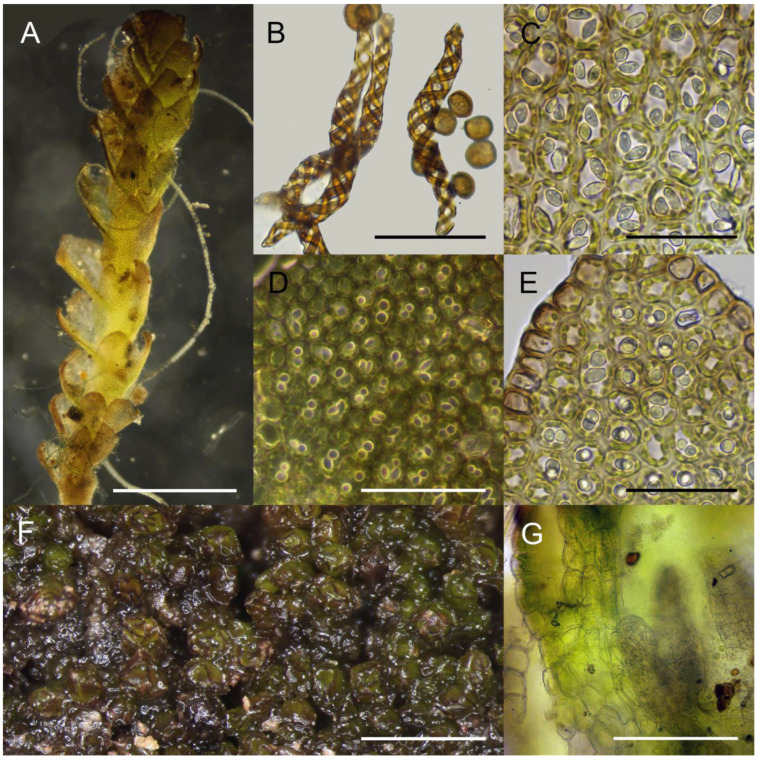
*Marsupella minutissima* N. Kitag.: (**A**) plant habit (photographed with dark field option); (**B**) elaters and spores; (**C**) midleaf cells with oil bodies; (**D**) cells in the lobe middle showing oil bodies (photographed with dark field option); (**E**) cells in the lobe middle showing oil bodies; (**F**) mat; (**G**) gynoecium and perigynium, longitudinal section, fragment showing some archegonia and perigynium wall. Scales: 500 µm for (**A**); 30 µm for (**B**,**C**,**E**); 50 µm for (**D**); 1000 µm for (**F**); 100 µm for (**G**). All from Kor-123-8-23 (VBGI, JNU).

**Figure 3:**
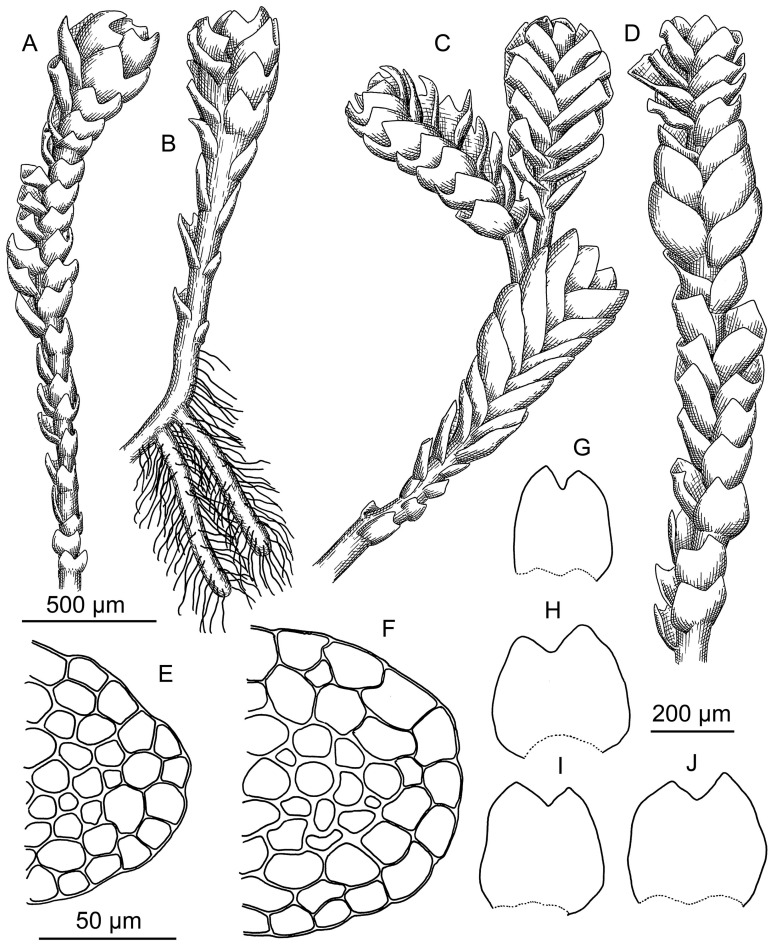
*Marsupella minutissima* N.Kitag.: (**A**) plant habit, dorsal view; (**B**) plant habit showing stolons, ventral view; (**C**) female plant, dorsal view; (**D**) male plant, dorsal view; (**E**,**F**) stem cross section fragment; (**G**–**J**) leaves. Scales: 500 µm for (**A**–**D**); 50 µm for (**E**,**F**); 200 µm for (**G**–**J**). All from Kor-122-2-23 (VBGI, JNU).

Plants in pure, rather dense patches, strongly incrusted by mineral (mostly small quartz particles) fine soil, erect, black to blackish brown in patches and blackish green when washed in the water and separated, with brownish bases (mostly decaying ones), (100–)200–350(–400) µm wide and 2–3 mm long. Rhizoids grayish, sparse in leaved shoot, where separated, obliquely spreading, while dense in geotropic stolons. Branching seen as ventral subfloral innovations, by 1–2 per one perichaetium, as well as common geotropic leafless and densely rhizogenous stolons. Stem cross section suborbicular to transversely ellipsoidal, ca 120 × 130 µm in well-developed shoots, with 1–2 layered hyalodermis composed by thin-walled cells 17–20 µm in diameter, with small trigones, in older part of shoot commonly erose; inner cells with somewhat thickened walls, 12–18 × 7–15 µm, with small, concave trigones; ventral side of stem sometimes brownish, in other parts colorless to greenish (chlorophyllose). Leaves subtransversely to slightly obliquely inserted, mostly imbricate, rarely contiguous and distant (in lower parts of shoot only), loosely sheathing the stem near base, obliquely spreading, subtransversely oriented, with margin commonly brownish colored, flattened in the slide well developed suborbicular to widely ovate, 260–280 × 280–370 µm, divided to 1/6–1/4 of the length by V- to ɣ-shaped sinus, in lower part of shoot commonly scale-like, less 100 µm in length and width. Midleaf cells subisodiametric to shortly oblong, 12–17 × 7–13 µm, thin-walled, trigones moderate in size, concave; oil bodies 2 per cell, finely granulate, oblong, 4–6 × 3–4 µm, mostly with hardly perceptible very small pupil; cuticle smooth. Dioicous. Male and female plants separated or sometimes intermixed within one patch. Androecia intercalary, spicate, where shoot 450–500 µm wide, with 2–3 pairs of bracts, shoot continuing growing above androecium as normal branch. Archegonia 5–7 per perichaetium. Perigynium 200–250 µm long, with 2 pairs of bracts, its wall 3 cell thick. Perianth rudimentary, widely conical, ca 100 µm high and 200 µm wide, hidden within bracts, composed by oblong thin-walled cells without or with vestigial trigones, unistratose, with crenulate to dentate (one-celled, cells thin-walled, oblong, but not acute). Innermost bract ca 300–400 µm long, with short sinus, outer bract 500–600 µm long, covering inner bract and the perianth. Capsule spherical, shortly emerging, with seta 500–700 µm long. Capsule wall bistratose, inner layer composed by rectangular (commonly irregularly so) cells 25–35 × 7–10(−20) µm, with ellipsoidal thickenings by 3–6 per longer wall and absent in short wall. Spores brown, finely verruculose, spherical 7–8 µm in diameter to shortly ellipsoidal, 10 × 8 µm. Elaters bispiral, rarely trispiral in the middle part, 60–100 × 8–10 µm, brown. (Figure 2 and Figure 3).

Specimens examined: Republic of Korea. Jeju-do. Gwangryeong-ri, Aewol-eup, Hallasan National Park, northwest slope of Hallasan Mountain: 33.36234° N 126.52002° E, 1612 m a.s.l. *Sasa quelpaertensis* meadow. Moist boulder near the trail. V.A. Bakalin & S.S. Choi (5 November 2023) Kor-122-1-23 (VBGI); 33.36249° N 126.52349° E, 1612 m a.s.l. Stream in narrow valley surrounded by dwarf *Sasa* and shrub community. Open moist stream boulder. V.A. Bakalin & S.S. Choi (5 November 2023) Kor-123-3-23, Kor-123-8-23 (VBGI); Mt. Hallasan, near top of mountain: 33.363601° N 126.526658° E, 1767 m a.s.l. *Sasa quelpaertensis* meadow. Moist boulder near the valley surrounded by *Abies koreana* community S.S. Choi (16 April 2022) 22,152 (JNU); Mt. Hallasan, near the top of the mountain. 33.363052° N 126.527638° E, 1787 m a.s.l. *Sasa quelpaertensis* meadow. S.S. Choi (6 November 2017) 170924, 170,963 (JNU); Japan. Shikoku, Ehime Prefecture, Mt. Nishiakaishi, leg K. Oti-1927 01 June 1947 (KYO, s.n., isotype of the taxon).

## 3. Discussion

### 3.1. Molecular Affinities

Most of the previously published phylogenies of *Marsupella* were based on a combination of nuclear ITS and plastid *trn*L-*trn*F or *trn*T-*trn*F regions [2,5,8,9], which provided phylogenetic signals that allowed species delimitation and the resolution of the affinities of species in this group. However, neither Porley et al. [5], who added a variable *trn*T-*trn*L spacer to the *trn*L-*trn*F plastid region, nor our study yielded suitable support and resolution of deep nodes within the sect. *Ustulatae*, where the target species *M. lusitanica* and *M. minutissima* are located. Additionally, indel coding did not improve resolution or nodal supports within the section. Further molecular phylogenetic studies of the backbone phylogeny of *Marsupella* can only be possible with the involvement of additional plastid markers, such as *trn*G and the whole *trn*S-*trn*F region, instead of only *trn*L-*trn*F. Conversely, the grouping of species might reflect phytogeographic patterns. In most versions of the obtained tree, specimens of *M. minutissima* did not form a supported clade with *M. disticha,* the single Japanese representative of the section included in the analysis; other clades were mostly composed of European or North Asian accessions. Therefore, East Asian representatives of this section require further examination.

### 3.2. Morphology

The above description is highly similar to the descriptions compiled from Japanese materials and current literature, with the exception of slightly larger cells in the middle of the leaf (12–17 × 7–13 µm in Korean populations, versus 13–15 × 11–14 µm in Kitagawa [7]) and medium-sized midleaf cell trigones (vs. indistinct in Kitagawa [7]). Oil bodies are described for this species for the first time and generally correspond to the *Marsupella* archetype, which are characterized, as a rule, by 2–3 oil bodies in the cells of the middle of the leaf, with a granulate surface. Deviations from this rule in other *Marsupella* taxa are rare.

*Marsupella minutissima* is placed in sect. *Ustulatae,* which has the most morphologically similar taxa. Two species most similar to *Marsupella minutissima* are *M. funckii* and *M. sprucei*. All three species are characterized by small erect plants, which usually do not exceed 5–8 mm in length, their patches are encrusted with mineral soil and commonly occur in locations with disturbed vegetation cover on bare fine soil. However, these species display special characteristics that make it possible to clearly distinguish one from another. The characteristics that help distinguish these taxa are listed in Table 1. Additionally, the three species are distinct in distribution, as discussed below. Among the possible ‘relatives’ of *M. minutissima*, Kitagawa [6] mentions *M. bolanderi* (belonging to sect. *Marsupella* in his paper, but now also considered to be within the sect. *Ustulatae*). However, *M. bolanderi* only very superficially (small size and blackish color of plants, patches often incrusted with mineral soil) resembles *M. minutissima*. *Marsupella bolanderi* can be immediately distinguished by the much larger cell size in the middle of the leaf (usually 20–30 µm in diameter) and more deeply divided leaves, up to 2/5 of the leaf length, usually with recurved lobes and a rounded lobe tips. The leaves of *M. bolanderi* appear as “smaller copies” of the leaves of *M. sphacelata* and are slightly similar to the leaves of *M. sprucei* var. *ustulata* (Spruce) Damsh., but with small cells in the middle of the leaf.

### 3.3. Ecology

Kitagawa [7] (p. 99) characterized the occurrence of the species quite briefly: “On rocks or soil under rather dry conditions at higher altitudes”. Our observations are fully consistent with the description provided. Although the specimens were all collected in a wet state (due to the rain at the day of collecting), the appearance of the habitat, with patches of bare soil and stones in open places under full sun, indicates that the habitats are often dry and very dry. Representatives of Pottiaceae or Grimmiaceae (the latter on rocks) often grow in such habitats. In the northern regions of Pacific Asia, similar habitats host *Marsupella sprucei* and *Gymnomitrion kamchaticum* Mamontov, Vilnet & Konstant. (on soil) or *G. concinnatum* (Lightf.) Corda and *G. pacificum* Grolle (on stones and rocks). Rocks in the sampling area in the Republic of Korea were clearly acidic, which is generally preferred by the vast majority of Gymnomitriaceae. In terms of environmental requirements, the species belongs to the group of mesoxerophytes or even xerophytes.

**Figure 4:**
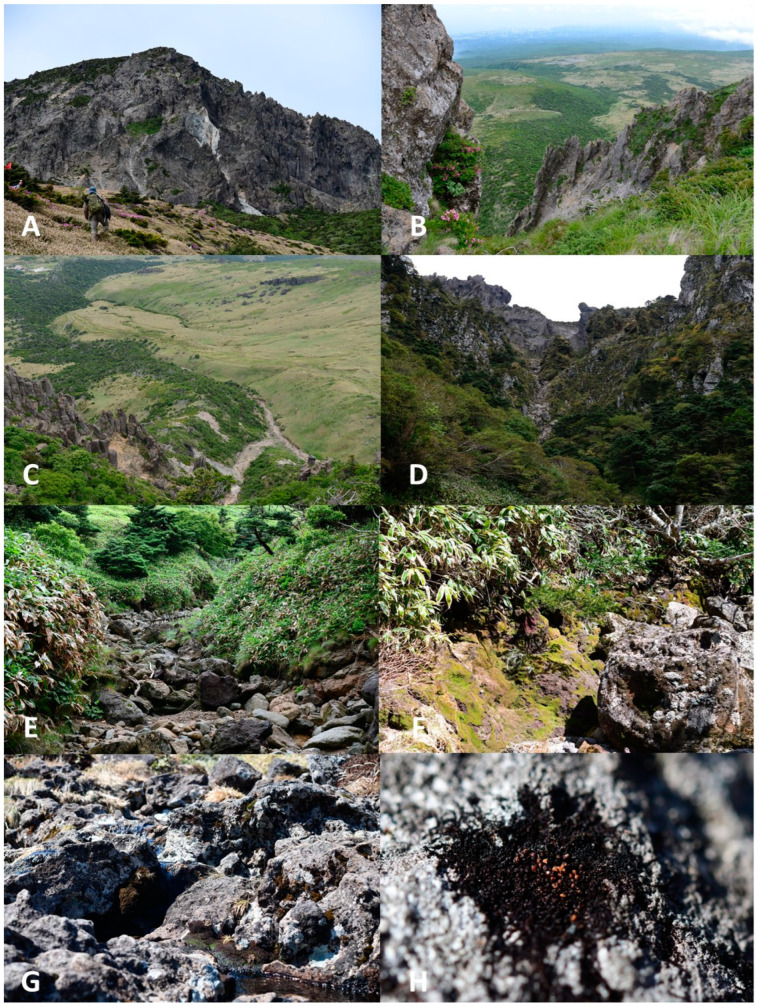
Habitat of *Marsupella minutissima*. (**A**). Baekrokdam Crater near the top of Hallasan Mountain; (**B**) Northwestern side of Baekrokdam Crater; (**C**) *Abies koreana* community near the starting point of Witse Oreum Valley; (**D**) Goanumsa Valley near the top of Hallasan Mountain; (**E**) Upper part of Witse Oreum Valley; (**F**). *Sasa quelpaertensis* community near the Witse Oreum Valley; (**G**) Natural habitat on rocks; (**H**) Mat of *Marsupella minutissima* covering rock. Photos by S.S.C., 2023.

In both the Republic of Korea and Japan, the thickets of *Sasa quelpaertensis* Nakai (often dwarf, up to 50 cm in height) is the dominant vegetation cover, with scattered clumps of shrubs (*Juniperus chinensis* var. *sargentii* A.Henry, *Weigela florida* (Bunge) A. DC.) and small spots of *Empetrum nigrum* subsp. *asiaticum* (Nakai ex H. Itô) Kuvaev. Spots of bare fine soil, due to erosion, and large boulders are present at each site. Though both collection points are located in the oro-boreal belt of the respective mountains [11], the sites are covered by intrazonal vegetation, and tall trees are absent. The habitats of the species are shown in Figure 4.

### 3.4. Distribution

Jeju-do is a remarkable location where taxa with both subtropical and arctomontane distributions meet each other. Significant elevation changes, landscape diversity, and community diversity make the island a hotspot for liverwort taxonomic diversity in East Asia. Our team previously reviewed the taxonomic composition and phytogeographical relationships of Jeju-do liverwort flora [11]. A total of 209 species of liverworts were known on the island (now 210, taking *Marsupella minutissima* into account), with the largest number of mountain taxa occurring near the peak of Hallasan Mountain. This area contains the type localities of at least three liverwort species: *Eocalypogeia quelpaertensis* (S. Hatt. & Inoue) R.M. Schust., *Solenostoma koreanum* Steph. (=*Protosolenostoma fusiforme* (Steph.) Vilnet & Bakalin, with some doubt) and *Solenostoma faurieanum* (Beauverd) R.M. Schust. Additionally, a number of globally rare species are known to occur the area surrounding the mountain summit, including *Gymnomitrion noguchianum* S. Hatt., and *Marsupella vermiformis* (R.M. Schust.) Bakalin & Fedosov. Moreover, the record of *Marsupella vermiformis* (R.M. Schust.) Bakalin & Fedosov, initially known from Melanesia, is the most distant and northernmost record worldwide. Although *Marsupella minutissima* was not found in the uppermost zone of Hallasan Mountain, it is probable that the species occurs in the area as it belongs to the aforementioned rare group of East Asian Mountain species. Choi et al. [11] described Jeju Island as a link for liverwort migrations between Japan and China during past marine regressions. *Marsupella minutissima*, along with other species, may have migrated across the former Japan-China land bridge, which encompassed Jeju-do. In general, the presence of *M. minutissima* on Jeju-do increases the likelihood species occurrence in the mountains of eastern China. Results of the molecular phylogenetic analyses suggest the high value of this East Asian floristic region as an epicenter of *Marsupella* sect. *Ustulatae* diversification, although the majority of taxa within it have montane North Holarctic distribution.

The morphological similarity of *Marsupella minutissima*, *M. funckii* and *M. sprucei* is discussed above. Although weakly differentiated morphologically, these species are easily distinguishable phylogenetically and by distribution. Of the three, *Marsupella minutissima* is the southernmost occurring species, and the only species found in the eastern part of the East Asian region. *Marsupella sprucei* is a circumpolar and mainly hemiarctic species, with a clear tendency to increase its frequency of occurrence in suboceanic regions. Its distribution in the highlands of the Sino-Himalaya in East Asia is not completely implausible but is unlikely. Although the species has been reported in China in the Xizang (Tibet), Sichuan and Yunnan Provinces [12], these reports need to be verified. For example, the recently described *Gymnomitrion sichuanicum* Bakalin & Vilnet is very similar to *Marsupella sprucei* but does not belong to *Marsupella* [13]. The third taxon, *Marsupella funckii*, is a hemiboreal-temperate montane and predominantly amphi-Atlantic species. In Europe, the ranges of *M. funckii* and *M. sprucei* formally overlap, but no direct competition has been observed since *M. funckii* occupies lower altitudinal levels below the timberline, whereas *M. sprucei* occurs above the timberline. Although there are reports of *M. funckii* in the middle and southern parts of the Russian Far East (from the Kamchatka Peninsula to the southern Kuril Islands), no specimens been tested or identified by molecular phylogenetic methods for the identity with European materials. Thus, the identity of the Atlantic and Pacific populations remains unknown.

## 4. Materials and Methods

### 4.1. Taxon Sampling

The specimens were collected from two locations on the northwestern macro-slope of Hallasan Mountain within Hallasan National Park on Jeju-do in November 2023. Orographically, Hallasan Mountain is the wide cone of a dormant shield volcano with numerous rock outcrops, parasitic craters, and ancient lava flows from the main cone. The volcano formed mainly in the Pliocene, and its highest peak is the highest point in the Republic of Korea—1947 m a.s.l. [14,15]. The specimens collected were studied for anatomy and morphology at the Team of National Ecosystem Survey of the National Institute of Ecology as well as at the laboratory of cryptogamic biota at the Botanical Garden Institute. Intravital *Marsupella* photographs were taken using digital cameras mounted on Olympus SZX16 and Olympus BX43 microscopes in the Botanical Garden-Institute laboratory. Observations of living plants provided information about oil bodies. In addition to the plants collected in November 2023, our team’s earlier collections from the same areas were reviewed. To clarify species identification, the isotype of the taxon from KYO was examined, as described below in the Specimens Examined section.

A molecular-phylogenetic assessment of the affinities of *Marsupella minutissima* was performed on the plants newly found in the Republic of Korea. The dataset for the phylogenetic analysis was compiled from the available *nr*ITS and plastid *trn*L-*trn*F sequences based on the datasets by Bakalin et al. [2] and Porley et al. [5]. Particular focus was given to the sect. *Ustulatae*, as this is the section to which the Korean plants were classified according to the BLAST search and results of brief analysis within the full Bakalin et al. [2] dataset. Representation of the other lineages of *Marsupella* was then limited, and accessions of *Gymnomitrion*, *Plectocolea* and *Metasolenostoma* were included as outgroups so that 52 accessions were included in the phylogenetic analysis. Within these 52, there were 42 accessions of *Marsupella* and 19 of sect. *Ustulatae*, including *M. bolanderi*, *M. disticha* Steph., *M. funckii* (F. Weber et D. Mohr) Dumort., *M. lusitanica* R.D. Porley & Jan Kučera, *M. neglecta* (Limpr.) Lindb., *M. sphacelata* (Giesecke ex Lindenb.) Dumort., *M. sprucei* (Limpr.) Bernet and two newly added specimens of *M. minutissima* (Table S1).

Laboratory protocol was the same as the protocol outlined in Bakalin et al. [2]. The newly acquired sequences were aligned using MAFFT v. 7.505 [16] with E-INS-i strategy. Unless otherwise stated, default settings implemented in CIPRES online portal were used [17], then manually edited in BioEdit [18]. Since the ingroup topologies inferred from the ITS (52 accessions, 896 positions) and *trn*L-*trn*F (52 accessions, 440 positions) data did not conflict, a combined dataset (52 accessions, 1336 positions) was analysed. Indels were coded using a simple indel coding approach [19] in SeqState 1.4.1 [20]. The combined dataset was divided into two partitions: one for the nuclear data and one for the plastid data. An additional partition was created for indels coded by binary code.

### 4.2. Phylogenetic Analyses

Bayesian analyses were performed by running two parallel analyses in MrBayes 3.2.7a [21]. The analyses consisted of six Markov chains and 5,000,000 generations, with a sampling frequency of one tree per 1000 generations. All analyses had the chain temperature set at 0.02 and utilized the GTR model with sampling throughout the model space (setting nst = mixed). Convergence of analyses was assessed via ESS values, which were checked using Tracer v.1.7.2. [22] and found to be higher than 200. Average deviation of split frequencies reached 0.01 after 1 mln generations. Consensus trees were calculated after omitting the first 25% of trees as burn-in. ML trees were computed in iQ-tree [23] via the web server with 1000 generations of ultrafast bootstrap and using the same model of nucleotide substitutions (GTR+G+I) and partitioning approach as the Bayesian analyses. Two accessions of the genus *Metasolenostoma* (Solenostomataceae) were used for rooting the tree.

## 5. Conclusions

This study examines the recent discovery of *Marsupella minutissima* in the Republic of Korea, which is the first known record of this species outside of Japan. This report confirms the close relationships between the mountain floras of Japan and insular Korea as well as the importance of Jeju-do as a connecting link between the two. This report also aims to discourage the presumed endemism of liverwort flora to Japan, a presumption which will continue to be disproved in the future as other species considered to be Japanese endemics are found in Korea or/and China. As molecular phylogenetic analyses show, this species belongs to the sect *Ustulatae*, not to the sect. *Marsupella*, as previously assumed. Three species from this section are particularly similar morphologically: *M. funckii*, *M. sprucei* and *M. minutissima*. Apart from genetic differences and a small number of peculiar morphological traits, the three species differ distinctly in distribution patterns. This distinction is not found in southern Korea nor Japan, where similar habitats are occupied by several species of the genus *Gymnomitrion* (from the same family as *Marsupella*), which belong to a clearly morphologically defined but phylogenetically valueless group of “*Apomarsupella*”.

## Figures and Tables

**Table 1:** Comparison of three morphologically similar species of *Marsupella* sect. *Ustulatae*.

Feature	*M. minutissima*	*M. funckii*	*M. sprucei*
Inflorescence	dioicous	dioicous	monoicous (paroicous)
Leaf spreading	narrowly spreading	widely spreading	appressed (spreading in its var. *ustulata*)
Leaf lobes	obtuse, not diverging	acute, diverging	obtuse to rounded, not diverging

## Data Availability

All data are contained within the article and Supplementary Materials.

## Supplementary Materials

The following supporting information can be downloaded at: https://www.mdpi.com/article/10.3390/plants13213037/s1, Table S1: GenBank accession numbers of and IDs of specimens included in the phylogenetic analysis. Originally studied specimens are in bold.
